# Disturbance regulates the density–body‐mass relationship of soil fauna

**DOI:** 10.1002/eap.2019

**Published:** 2019-12-02

**Authors:** Frank van Langevelde, Vincent Comor, Steven de Bie, Herbert H. T. Prins, Madhav P. Thakur

**Affiliations:** ^1^ Resource Ecology Group Wageningen University Droevendaalsesteeg 3a Wageningen 6708 PB The Netherlands; ^2^ School of Life Sciences University of KwaZulu‐Natal Westville Campus Durban 4000 South Africa; ^3^ Department of Terrestrial Ecology Netherlands Institute of Ecology (NIOO‐KNAW) Droevendaalsesteeg 10 Wageningen 6708 PB The Netherlands

**Keywords:** abundance–mass relationship, biomonitoring, community ecology, population density, recovery, restoration, scaling laws

## Abstract

Theory on the density–body‐mass (DBM) relationship predicts that the density of animal species decreases by the power of −0.75 per unit increase in their body mass, or by the power of −1 when taxa across trophic levels are studied. This relationship is, however, largely debated, as the slope often deviates from the theoretical predictions. Here, we tested the ability of the DBM relationship to reflect changes in the structure of communities subjected to an anthropogenic disturbance. The slope would become less steep if smaller animals were more impacted by the disturbance than the larger ones, whereas the slope would become steeper if larger animals were more affected than the smaller ones. We tested the changes in the DBM relationship by sampling soil fauna, i.e., nematodes, Collembola, and larger arthropods, from a semiarid grassland before and after spraying diesel fuel as disturbance. We applied three different treatments: a control, a light disturbance, and an intense disturbance. We found that the slopes of the DBM relationships before the disturbance were around −1 as predicted by theory. The slope became more positive (i.e., less steep) just after the disturbance, especially after the intense disturbance as smaller fauna suffered the most and early colonizers had larger body mass. Interestingly, we observed that the slopes converged back to −1 by 2 months post‐disturbance. Our findings show that the response of soil fauna communities to anthropogenic disturbances could explain the large variation in observed slopes of the DBM relationships. We experimentally demonstrate that an animal community, when disturbed, shows a temporal pattern of DBM relationships ranging from deviations from the predicted slope to convergence to the predicted slope with time. We recommend that deviations in the DBM relationships after disturbances can provide insights in the trajectory of community recovery, and hence could be used for biomonitoring.

## Introduction

Understanding the patterns of species density is a key challenge in ecology (Gaston and Blackburn 2000, Rosenzweig et al. 2008). There is extensive empirical evidence for negative scaling of the population density of species in relation to their body size, i.e., the density (*D*)–body‐mass (*M*) relationship (hereafter referred to as the DBM relationship; Elton 2001, Mohr 1940, Damuth 1981, 1987, Duarte et al. 1987, Enquist et al. 1998). Theory predicts a negative DBM relationship described as the power law *D = a* × *M* ^*b*^, where *a* is the normalization constant and the exponent *b* is approximately −0.75 for taxa within a single trophic level (Nee et al. 1991, West et al. 1997, Brown et al. 2004). The same exponent *b* is reported to have the value of −1 when taxa across trophic levels are studied (Peters and Wassenberg 1983, Boudreau and Dickie 1992, Schmid et al. 2000). However, these two exponents are not consistently found (White et al. 2007) and even positive DBM relationships (Russo et al. 2003, Maxwell and Jennings 2006) or no relationship (e.g., Gaston and Lawton 1988) have been observed. Moreover, DBM relationships can also be nonlinear, such as of a polygonal shape (Brown and Maurer 1987, Cotgreave 1993, Leaper and Raffaelli 1999, Andrew and Hughes 2008). The inconsistent DBM relationships are attributed to several explanations, such as a narrow range of body size of the study taxa (Brown and Maurer 1987, Morse et al. 1988, Silva and Downing 1994, Cyr et al. 1997a), sampling artefacts (Lawton 1989, Arneberg and Andersen 2003), and sampling restricted to a single taxon (Schmid et al. 2000). Turnbull et al. (2014) suggested that changes in the DBM relationships might be a community response to changes in the environmental conditions. Several studies have indeed shown that the DBM relationships vary between different environmental conditions (e.g., Xu et al. 2015, Zhao et al. 2015). In this paper, we show that differences in community composition in response to changes in environmental conditions can explain inconsistency in the DBM relationship.

When a community is subjected to environmental modifications due to disturbances, its DBM relationship is expected to change because of the shifts in density and species composition (and hence, changes in body mass distribution) of the community (Cyr et al. 1997b, Siqueira et al. 2008, Reuman et al. 2009). For instance, some species may withstand the disturbance, whereas others may decrease in density or even disappear locally. Moreover, the ability to reproduce fast, a high tolerance to disturbance agents, a particular diet or fast dispersal capabilities are traits that allow organisms to (re)colonize a disturbed system (Thakur et al. 2014). For instance, recent studies have pointed that large‐sized organisms can have movement advantage during their dispersal albeit until a threshold body mass (Hirt et al. 2017, 2018). Given that many of these traits are related to body mass, the DBM relationship of such a disturbed system may deviate from its predicted slope. We experimentally tested the effect of an anthropogenic disturbance on the slope of the DBM relationship of soil fauna communities by covering a large gradient in animal body mass and monitored the community composition before and after the disturbance.

When a disturbed soil animal community returns to its initial state, which is at least functionally similar (Bengtsson 2002), we expect that the slope of the DBM relationship in a disturbed soil fauna community would have a different slope prior to the disturbance, and that the slope would return to its initial value as the community progressively recovers. Depending on the nature and severity of the disturbance, different body mass categories of the soil fauna communities may be more impacted than others. If smaller animal species suffered more from the disturbance than larger ones, then the slope would be more positive after the disturbance (less steep slope; Cyr et al. 1997a, Comor et al. 1993), whereas the slope would be more negative (steeper slope) if larger animal species were more impacted than smaller ones (Reuman et al. 2009). The variation in the effects of a disturbance on different size classes of member species within a community is crucial for predicting community structure and functioning after the disturbance (Simon 1976, Sherry and McDade 1982, Pearson and Derr 1986, Greenberg and McGrane 1996). We sampled a wide range of soil fauna including nematodes, Collembola, and larger arthropods, spanning nearly seven orders of magnitude in their body mass. In order to create a disturbance that would allow for a rapid recovery, we used diesel fuel. While diesel is toxic to these animals (The Shell Company of Australia Ltd 2010), it has minimal effects on their habitat, such as the availability of resources (Comor 2013). Further, as diesel is relatively volatile, we excluded the possibility of long‐term toxic effects in our experiment. Accordingly, nematodes, Collembola and larger soil arthropods are known to recover after this disturbance (Rosenberg et al. 1986, Brmez et al. 2008, Zeppelini et al. 2009).

## Methods

### Experimental design

Field work took place in Wits Rural Facility, Hoedspruit, Limpopo Province, South Africa (24°15′20.23″ S, 31°23′23.63″ E) during the wet season from November 2009 until January 2010. The area is covered with savanna vegetation and is part of the Granite Lowveld region. The average climate of the area is classified as semiarid under the Köppen‐Geiger System (Kottek et al. 2006). The long‐term mean yearly rainfall is about 438 mm, the mean maximum temperature during January (hottest month) is 33.7°C and the mean minimum temperature in June (coolest month) is 9.4°C.

We chose 10 blocks at least 10 m away from one another so that they shared similar vegetation and soil type: grassland, no shrubs or trees, 30 cm sand on top of the clay layer. Each block had three plots of 3 × 3 m, separated by at least 5 m, with different treatments: a control, a light disturbance, and an intense disturbance (Appendix S1: Fig. S1). The disturbance was created by spraying diesel fuel with a backpack sprayer onto the surface of the ground, with doses known to impact soil fauna (Comor 2013): 100 mL/m^2^ for the light disturbance and 200 mL/m^2^ for the intense disturbance. In these plots, we sampled soil fauna four times: 3 weeks before the disturbance, 1 d after the disturbance, and again 1 month and 2 months after the disturbance.

To sample litter and epigeous arthropods, five pitfall traps per plot, 13 cm deep and 9 cm wide (to decrease the bias toward high‐body‐mass species, Ulrich et al. 2005) were opened for 3 d at each sampling event, with 2 cm of salt‐saturated water as a preservative. These pitfall traps were evenly distributed in each plot so that they each covered an area of around 2 m^2^. We assumed that the time between the sampling events allowed the arthropods to recolonize each of the 2‐m^2^ areas. Collembola were sampled by collecting four cores per plot of the first 5 cm of the soil with a 4.1‐cm corer. The locations differed between the sampling events (at least 50 cm distance to reduce the effects of removing soil in the locations that were earlier used). Samples were then extracted in Tullgren funnels (Van Straalen and Rijninks 1982) for 2 weeks. Tullgren funnels were 10 cm in diameter and ~5 cm high with animal extractions obtained using a temperature gradient from 30°C at the top and 5°C at the bottom. Two orders of Collembola were identified, Entomobryomorpha and Poduromorpha. Nematodes were sampled by collecting six cores of soil per plot with a 1.5 cm wide corer to a depth of 10 cm. Again, the locations differed between the sampling events. These samples were gently mixed together and 120 mL of soil was used for the subsequent extraction, following Cobb's method with a sieve set with the mesh sizes 1,000, 355, 175, 100, and 45 μm (Cobb 1918, Van Bezooijen 2006). The debris from the last four sieves was collected and gently poured on a round filter (20 cm wide) with a small water column beneath it. The filter and water column were closed with a lid and kept in the dark at 18–20°C. The nematodes had 48 h to move into the water column. The nematodes were harvested from the water column and counted.

### Body mass determination

The animals collected in the pitfall traps were soaked in water and rinsed to eliminate the salt, dried at 70°C for 48 h, and weighed with a microscale (precision of 1 μg). The Collembola and nematodes were categorized visually into two classes based on their body size: large and small size. For each of these groups of Collembola and nematodes, 100 individuals, regardless of species, were dried at 70°C for 48 h and weighed together (each group separately) with the same microscale to obtain an estimate of the average mass of one individual per group. For the Collembola, the mass per individual was 43 μg for the small size class and 71 μg for the large size class. For the nematodes, the mass per individual was 0.21 μg for the small size class and 0.38 μg for the large size class. For both Collembola and nematodes, the weights correspond to what has been reported in the literature (Fjellberg 1998, 2007, Tita et al. 1999).

Body mass classes of nematodes, Collembola, and larger arthropods were defined based on the log_10_(body mass in grams). To maximize the representation of the different size classes, we were able to have 16 classes ranging from −6.74 to 0.50. Each class corresponded to one‐quarter of one log_10_(body mass in grams) unit, with the first class being −6.74 to −6.50, then −6.49 to −6.25, and so on until the last class 0.24 to 0.50. All nematodes belonged to the first class, Collembola to the class −4.24 to −4.00, and the animals caught in the pitfall traps to the remaining classes. For two size classes, we did not find any individuals (see details in Appendix S2: Table S1).

### Calculation of densities

Total abundances of animals were converted into densities (number of individuals/m^2^) to allow for comparisons. For each sampling event, total abundances of all the pitfall traps of each treatment (5 pitfall traps × 10 plots = 50 pitfall traps) were divided by the sampling area, i.e., 90 m^2^ (10 plots of 9 m^2^), that was considered to be sufficiently covered by the pitfall traps. We could not use fences around the plots to obtain absolute density estimates because of the large herbivores roaming in the area, which would have destroyed these fences, and to prevent depleting the arthropods in the plots (see *Statistical analyses* for the verification of the size of the sampling area).

The abundances of Collembola and nematodes were not considered relative to a volume, but to a surface area, since most of these animals live in the first few centimeters of the soil. The total abundance of Collembola of the four samples was translated into the number of individuals of Collembola per m^2^ based on the surface area of the corer. The density of the nematodes was calculated using the volume of soil that was used for the extraction and related to the surface area of the cores.

### Statistical analyses

The slopes of the DBM relationships were estimated using maximum likelihood estimates as recommend by White et al. (2007). Density was used as a dependent variable and we tested the effect of body mass, disturbance (as a categorical variable) and the interaction between body mass and disturbance on density. We used the individual size distribution (ISD) or size spectrum to analyze the slope of the DBM relationship (Reuman et al. 2008) as this is one of the most commonly used approaches in DBM studies (White et al. 2007). ISDs describe the pattern relating the number of individuals in a body‐size class (irrespective of species) and the average size of that body‐size class. The maximum likelihood estimates were obtained from using the *bbmle* package (Bolker 2017) of the R statistical software (R Core Team 2018). The pseudo *R* ^2^ (McFadden's *R* ^2^) for the DBM slopes was obtained using the *DescTools* package (Signorell et al. 2016). Furthermore, we also used reduced major axis regression (Type II regression) (Legendre and Legendre 1998) to calculate the DBM slopes using the *lmodel2* package (Legendre 2018) and compared those with the slopes obtained from maximum likelihood estimates. All slope calculations and statistical analyses were carried out separately for each sampling event.

**Table 1:** Effects of disturbance on the density of soil fauna with their body mass as covariate and the interaction between disturbance and body mass

Sampling time points	Disturbance			Body mass			Disturbance × body mass		
	*F*	df	*P*	*F*	df	*P*	*F*	df	*P*
Before disturbance	0.22	2,35	0.80	**264.95**	1,35	**<0.001**	<0.01	2,35	0.99
Day 1	**5.21**	2,36	**0.01**	**609.74**	1,36	**<0.001**	**6.61**	2,36	**<0.01**
Day 30	1.16	2,35	0.32	**367.49**	1,35	**<0.001**	**6.41**	2,35	**<0.01**
Day 60	0.36	2,34	0.69	**220.12**	1,34	**<0.001**	1.64	2,34	0.20

Notes

The *F* and *P* values are obtained from the F tests (Type II sum of squares) on linear models using the *car* package (Fox and Weisberg 2011) in R. Significant (*P* ≤ 0.05) values are highlighted in boldface text.

In order to verify the validity of the assumption that the sampling area for calculating the densities of the arthropods covered 9 m^2^ (3 × 3 m plots), we also computed the DBM relationships with the same total abundances but now with plot sizes of 6 and 12 m^2^, to estimate the variation between the slopes of the DBM relationships (the densities of nematodes and Collembola were kept the same for each calculation). There were no differences between the slopes obtained for the three sampling areas 6, 9 and 12 m^2^ (results not shown), therefore we chose to use the densities of pitfall trap arthropods based on 9 m^2^ for the analyses.

## Results

**Figure 1:**
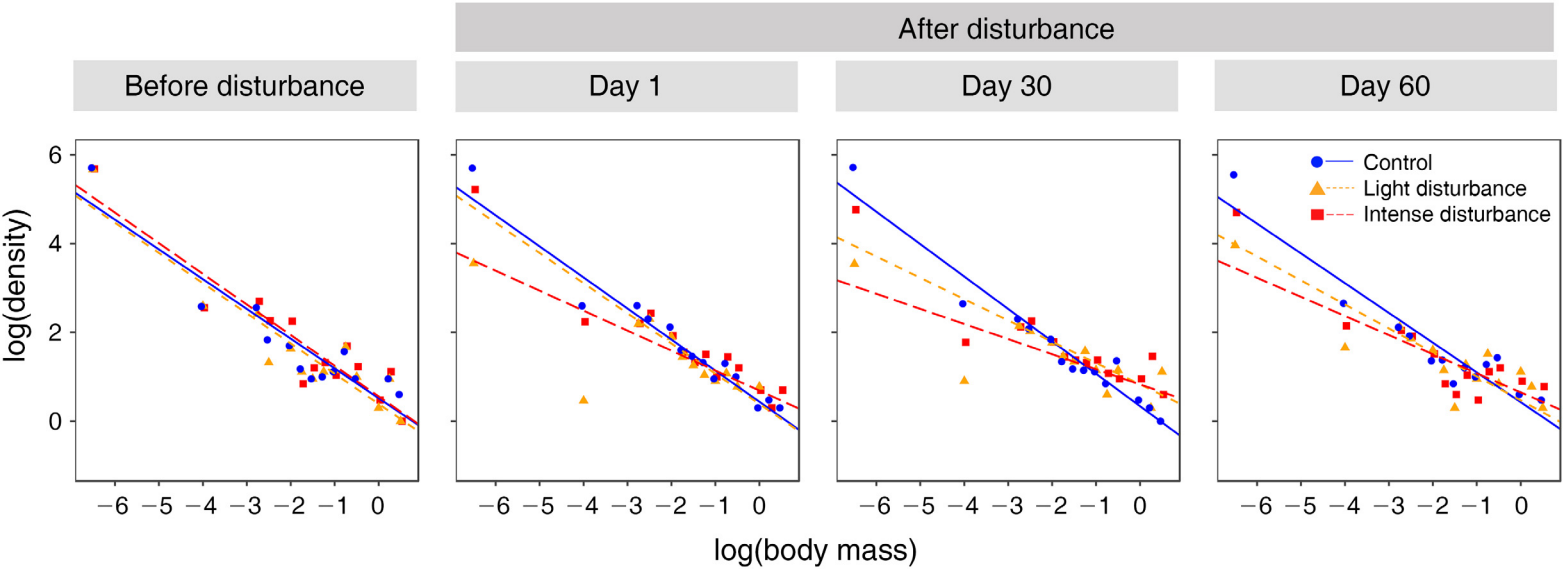
Density–body‐mass (DBM) relationship of all the animals collected in the three treatments and at different sampling time points before and after the disturbance. Density was measured as no. individuals/m^2^; body mass was measured as g.

**Figure 2:**
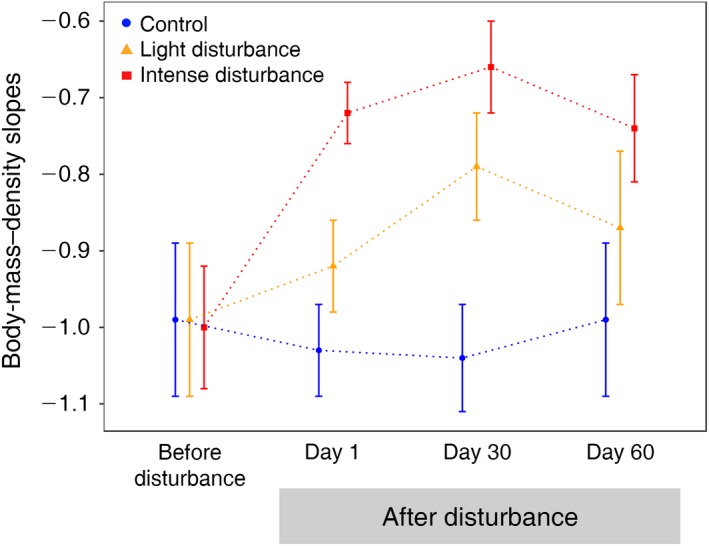
Changes in the slope of the DBM relationship (±SE) for the three treatments of disturbance before and after the application of disturbance. The details of the slopes are provided in Table 2.

A total of 22,535 nematodes, 721 Collembola, and 6,575 arthropod individuals from the pitfall traps were collected, from which the densities of each body‐mass class were determined (Appendix S2: Table S1). Before the disturbance, the slopes of the DBM relationships of the three treatments were almost perfectly parallel, with slopes around −1 that did not differ significantly between each other (Figs. 1 and 2). Just after the disturbance (day 1), the slope of the DBM relationship of the intensely disturbed plots increased to −0.72, which was significantly higher than the lightly disturbed and the control plots (Fig. 2, Table 2). The slope of the lightly disturbed plots increased to −0.92 and the slope of the control plots remained similar, but these were not significantly different (Figs. 1 and 2). Thirty days after the disturbance, the slopes of the disturbed plots continued to increase, with the intensely disturbed one reaching −0.66 and the lightly disturbed one −0.79, while the control slope remained around −1 (Figs. 1 and 2, Table 2). Both slopes of the disturbed plots were significantly higher than the control after 30 d from the introduction of disturbance (Table 2). Only after 60 d did the slopes of the disturbed plots started to decrease (Fig. 1). The DBM slopes did not vary anymore between the treatments and converged again to −1 (Table 1). The slopes obtained from Type II regression matched the results from maximum likelihood estimates (Appendix S3: Table S1, Fig. S1).

**Table 2:** The disturbance–body‐mass (DBM) slopes from linear models using maximum likelihood estimations

Sampling time point and disturbance	Slope	SE	Log‐likelihood	Pseudo *R* ^2^
Before disturbance				
Control	−0.99	0.1	26.45	0.50
Light	−0.99	0.1	26.45	0.49
Intense	−1.00	0.08	24.97	0.56
Day 1				
Control	−1.03	0.06	14.02	0.75
Light	−0.92	0.06	17.13	0.68
Intense	−0.72	0.04	6.67	0.85
Day 30				
Control	−1.04	0.07	19.96	0.65
Light	−0.79	0.07	20.65	0.59
Intense	−0.66	0.06	11.50	0.73
Day 60				
Control	−0.99	0.1	25.29	0.52
Light	−0.87	0.1	25.50	0.49
Intense	−0.74	0.07	21.87	0.55

SE stands for standard error of the slope. The log‐likelihood and pseudo *R* ^2^ are shown as model fits. The listed log‐likelihood values are multiplied by −2 as in the *bbmle* package (Bolker 2017) in R.

At day 1 (just after the disturbance), the density of Collembola (with a log_10_(body mass) of −4) dropped proportionally more than the densities of animals of other body mass classes (Fig. 1, Appendix 2). The largest arthropods present in the intensely disturbed plots before the disturbance (Fig. 1) appeared to be less abundant immediately after the disturbance (Fig. 1). However, these large arthropods were in higher number after a month (Fig. 1), thereby contributing in making the DBM slope steeper. These results also match with the patterns observed for changes in density of two body‐size classes of nematodes and Collembola as well as for various body‐size classes of arthropods (Appendix S4: Fig. S1, Appendix S5: Fig. S1 and Appendix S6: Fig. S1).

## Discussion

In this study, we experimentally tested the effect of an anthropogenic disturbance on the slope of the DBM relationship of soil fauna communities. Our study demonstrates the changes in the DBM relationships in response to changes in environmental conditions, which is supported by several studies (George and Lindo 2015, Xu et al. 2015, Zhao et al. 2015). Although the DBM relationship at local scales can be highly variable (White et al. 2007), we found values for the slope between −1.04 and −0.99 in the control communities, as expected for communities that contain several trophic levels (Peters and Wassenberg 1983, Boudreau and Dickie 1992, Schmid et al. 2000) over our observation period. In accordance with our hypothesis, the intense disturbance caused greater deviations of the slope of the DBM relationship than the light disturbance, and both began to converge with the control as the community recovered from the disturbance. Our results thus highlight that variations in the DBM relationship could be due to the response of animal communities to changing environmental conditions, such as related to disturbances that can locally eliminate animals of a given body size (Turnbull et al. 2014). The response of animal communities to changing environmental conditions, such as anthropogenic disturbances, could therefore explain the large variation in observed slopes of the DBM relationships.

Contrary to studies on a narrow range of animals or a single taxon (Brown and Maurer 1987, Morse et al. 1988, Silva and Downing 1994, Russo et al. 2003, Ulrich et al. 2005), our data contained several trophic levels in soil fauna thanks to the use of different sampling methods, and thus with several orders of magnitude of body mass. Moreover, different sampling methods to capture soil animals minimized a bias toward capturing only the most abundant species (Lawton 1989). As we sampled the local communities several times in the same area, our approach yielded a higher chance to catch the rare species (Blackburn et al. 1993) resulting into a more robust DBM relationship, but also with a greater sensitivity to changes in environmental conditions (Stork and Blackburn 1993, Cyr et al. 1997a,b). Our study further highlights that a greater temporal resolution of the DBM relationship can help understand temporal variations associated with community characteristics (Cyr et al. 1997b), given we observed a temporal convergence of size structure within the soil communities after the disturbance event.

Our results show that smaller (intermediate sized in this study) animals decreased more from the disturbance than larger ones (i.e., larger arthropods) resulting in a more positive slope after the disturbance (shallower slope; Cyr et al. 1997a). This higher positive slope of the disturbed communities confirms previous findings that steeper (more negative) slopes usually characterize communities living in a more stable environment (Cyr et al. 1997a, Jennings and Mackinson 2003). The question remains whether decrease in the density of the smaller animals is a common response to disturbances among soil fauna (e.g., Bokhorst et al. 2012, Gibb et al. 2018) and if so can it be used as a common indicator of disturbance (e.g., Niklaus et al. 2003, Lindo et al. 2012, Andriuzzi et al. 2017). The smaller animals, especially the Collembola, seem to have been more impacted just after the disturbance than the other groups of animals after the diesel fuel was added (Fig. 1). In fact, we also observed that proportional decline in both smaller nematodes and smaller Collembola were the highest immediately after the disturbance compared to other size groups (Appendix S4: Fig. S1, and Appendix S5: Fig. S1). Although Collembola largely differ in their preferred soil depth and dispersal ability, this impact could be explained by the fact that they live very close to the surface and are known to be organisms sensitive to disturbances, often used as biological indicators in polluted areas (Zeppelini et al. 2009). This seemed mainly true for smaller Collembola groups when we explored the density changes post‐disturbance between the small and large Collembola (Appendix S5: Fig. S1). Interestingly, nematodes have also been used as bioindicators, owing to their high sensitivity to disturbances (Bongers and Bongers 1998); changes in their density further relates to moisture and sand content in the soil (Hunt et al. 2001, Neher 2010, Thakur et al. 2017). The slope of the DBM relationship may have increased by Day 30 after disturbance because larger soil organisms having better dispersal capabilities began to colonize the site (Brown et al. 2004, Jenkins et al. 2007, Hirt et al. 2017). We thus suspect that the positive slopes during the recovery period could be related to the greater dispersal ability of larger arthropods compared to their recovery from faster reproduction after the disturbance.

As body mass of species is related to many other traits (Peters 1983, Peterson et al. 1998, Lewis et al. 2008), such as physiological, behavioral, and ecological characteristics of an animal, it is often suggested that body mass could improve predictive capabilities for biomonitoring (Cyr et al. 1997b, Layman et al. 2005). When the DBM relationship follows a trajectory temporal after a disturbance, as was the case in our study, the dynamics of DMB relationships can be used as an indicator of the state and health of an ecosystem, particularly when related to ecosystem recovery at the community level. Furthermore, both density differences and body mass spectra are valuable in comparing large‐scale structural patterns among ecological communities (because they encompass information both on the size of organisms and on the community biomass), they are intimately tied to ecosystem functioning (Cyr et al. 1997b, Brown et al. 2004). Other experimental studies also support the idea to use the DBM relationship in soil assessments after disturbances (George and Lindo 2015). We thus encourage future studies examining the recovery of ecosystems, such as in restoration research, to incorporate the temporal aspects of DBM relationships of animal communities across trophic groups.

## Data Availability

Data are available from the Dryad Digital Repository: https://doi.org/10.5061/dryad.f7m0cfxrb
