# Supplementary material for: Disturbance regulates the density–body‐mass relationship of soil fauna
Source: Ecol Appl. 2019 Dec 2;30(1):e02019. doi: 10.1002/eap.2019 (PMC7003476; doi:10.1002/eap.2019)
Supplement: Supplementary file 1 [file EAP-30-e02019-s001.pdf]

Supporting Information. Frank van Langevelde, Vincent Comor, Steven de Bie, Herbert H. T. Prins, Madhav P. Thakur. 2019. Disturbance regulates the density–body mass relationship of soil fauna. *Ecological Applications*.

Appendix S1

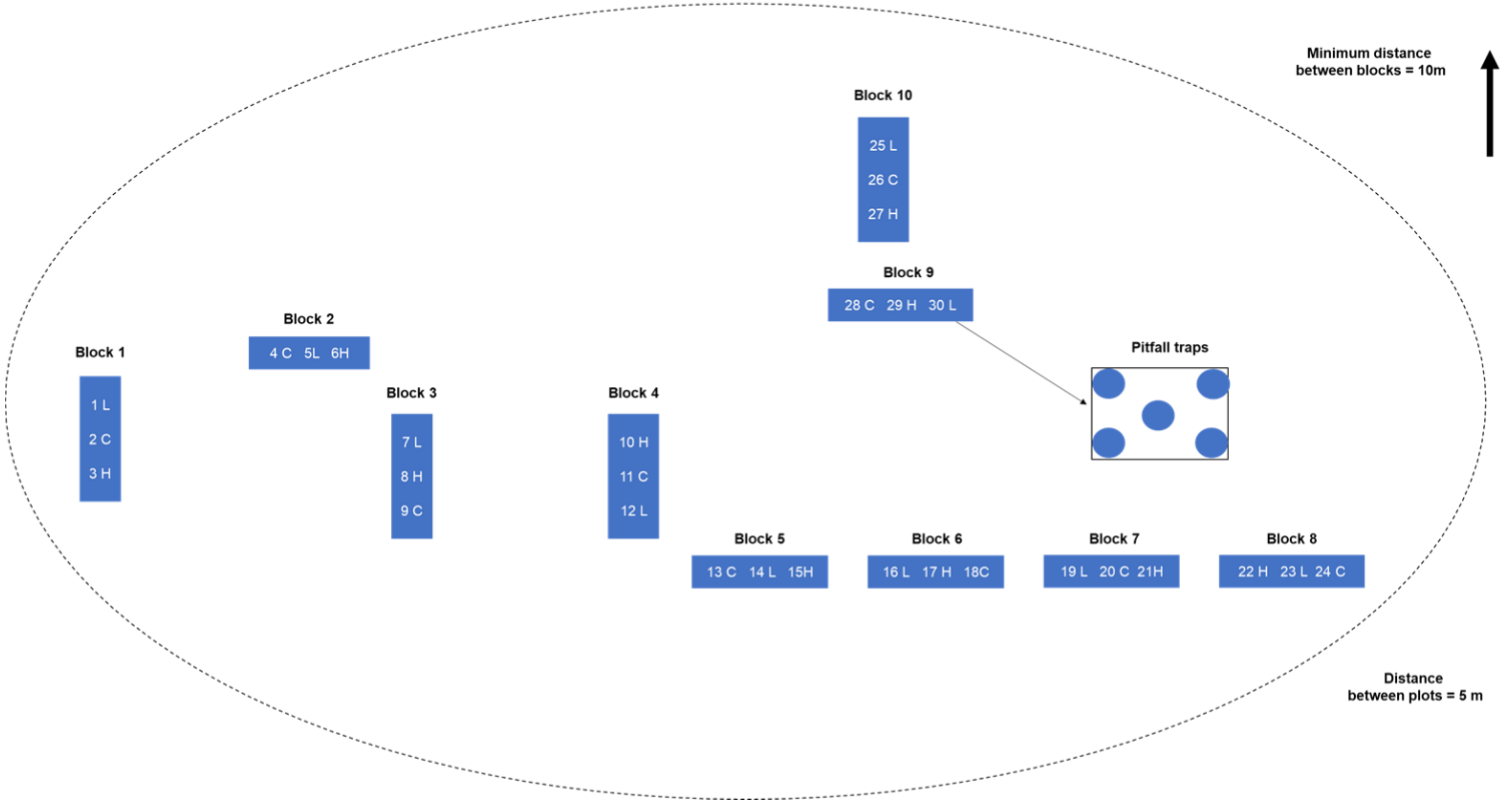

Figure S1: An overview of experimental design. Within each block, we had three treatments with a gradient of disturbance (C-control, L-lighter intensity of disturbance, and H-high intensity of disturbance). Pitfall traps were placed in each plot in orientation shown in the figure. The distance between blocks are based on a rough estimate and should only be used for a general idea.
