# Supplementary material for: Disturbance regulates the density–body‐mass relationship of soil fauna
Source: Ecol Appl. 2019 Dec 2;30(1):e02019. doi: 10.1002/eap.2019 (PMC7003476; doi:10.1002/eap.2019)
Supplement: Supplementary file 2 [file EAP-30-e02019-s002.pdf]

Supporting Information. Frank van Langevelde, Vincent Comor, Steven de Bie, Herbert H. T. Prins, Madhav P. Thakur. 2019. Disturbance regulates the density–body mass relationship of soil fauna. *Ecological Applications*.

Appendix S2

Table S1: Densities of animals (number of individuals per m^2) according to their body mass class for each sampling and treatment. Bottom table: log-transformed values (log_{10}) of the densities and their corresponding log-transformed (log_{10}) body mass classes. Numbers have been rounded to the second decimal in both tables, but analyses were performed on raw numbers. The animals caught included Araneae, Blattodea, Chilopoda, Coleoptera, Diplopoda, Formicidae, Hemiptera, Mantodea, Oniscidea, Orthoptera, Phasmatodea, Scorpiones and Solpugida.

Sampling	Treatment	Nematodes	Collembola	Pitfall trap arthropods													
A)		0.18μg to 0.32μg	57.5μg to 100μg	1mg to 1.78mg	1.79mg to 3.16mg	3.17mg to 5.62mg	5.63mg to 10mg	10.1mg to 17.8mg	17.9mg to 31.6mg	31.7mg to 56.2mg	56.3mg to 99.9mg	0.1g to 0.18g	0.19g to 0.32g	0.33g to 0.56g	0.57g to 1g	1.01 to 1.78g	1.79g to 3.16g
3 weeks before	Control	521000	440	4.03	0.76	-	0.56	0.17	0.10	0.11	0.14	0.41	0.10	-	-	0.10	0.04
	Light	487167	412	2.92	0.23	-	0.48	0.14	0.10	0.14	0.16	0.52	0.11	-	0.02	0.10	0.01
	Intense	493167	388	5.58	2.02	-	1.98	0.08	0.18	0.23	0.12	0.54	0.19	-	0.03	0.14	0.01
1 day after	Control	530000	431	4.46	2.22	-	1.47	0.44	0.32	0.23	0.10	0.22	0.11	-	0.02	0.03	0.02
	Light	175833	213	1.77	3.02	-	0.94	0.40	0.23	0.36	0.11	0.31	0.18	-	0.06	0.02	0.06
	Intense	3600	3	1.74	2.24	-	0.88	0.31	0.20	0.12	0.09	0.13	0.07	-	0.07	0.01	0.01
1 month after	Control	537800	492	2.23	1.34	-	0.78	0.24	0.17	0.16	0.14	0.08	0.26	-	0.03	0.02	0.01
	Light	58258	156	1.49	1.99	-	0.68	0.31	0.27	0.23	0.27	0.13	0.10	-	0.10	0.32	0.04
	Intense	3500	8	1.54	1.19	-	0.66	0.32	0.24	0.42	0.16	0.04	0.16	-	-	0.02	0.14
2 months after	Control	380667	497	1.46	0.92	-	0.26	0.27	0.08	0.18	0.11	0.21	0.30	-	0.04	-	0.03
	Light	50285	166	1.23	0.91	-	0.37	0.08	0.04	0.12	0.03	0.14	0.18	-	0.09	-	0.07
	Intense	9200	99	1.47	0.81	-	0.44	0.16	0.02	0.21	0.10	0.37	0.08	-	0.14	0.07	0.02
B)		-6.74 to -6.5	-4.24 to -4	-3 to -2.75	-2.74 to -2.5	-2.49 to -2.25	-2.24 to -2	-1.99 to -1.75	-1.74 to -1.5	-1.49 to -1.25	-1.24 to -1	-0.99 to -0.75	-0.74 to -0.5	-0.49 to -0.25	-0.24 to 0	-0.01 to 0.25	-0.26 to 0.5

3 weeks before	Control	5.72	2.64	0.61	-0.12	-	-0.26	-0.78	-1.00	-0.95	-0.84	-0.39	-1.00	-	-	-1.00	-1.35
	Light	5.69	2.61	0.47	-0.63	-	-0.32	-0.84	-1.00	-0.84	-0.81	-0.28	-0.95	-	-1.65	-1.00	-1.95
	Intense	5.69	2.59	0.75	0.31	-	0.30	-1.11	-0.75	-0.63	-0.91	-0.26	-0.72	-	-1.48	-0.84	-1.95
1 day after	Control	5.72	2.63	0.65	0.35	-	0.17	-0.35	-0.49	-0.63	-1.00	-0.65	-0.95	-	-1.65	-1.48	-1.65
	Light	5.25	2.33	0.25	0.48	-	-0.02	-0.40	-0.63	-0.45	-0.95	-0.51	-0.75	-	-1.26	-1.65	-1.26
	Intense	3.56	0.48	0.24	0.35	-	-0.06	-0.51	-0.70	-0.91	-1.05	-0.88	-1.18	-	-1.18	-1.95	-1.95
1 month after	Control	5.73	2.69	0.35	0.13	-	-0.11	-0.61	-0.78	-0.81	-0.84	-1.11	-0.59	-	-1.48	-1.65	-1.95
	Light	4.77	2.19	0.17	0.30	-	-0.17	-0.51	-0.57	-0.63	-0.57	-0.88	-1.00	-	-1.00	-0.49	-1.35
	Intense	3.54	0.90	0.19	0.08	-	-0.18	-0.49	-0.61	-0.37	-0.81	-1.35	-0.81	-	-	-1.65	-0.84
2 months after	Control	5.58	2.70	0.16	-0.04	-	-0.59	-0.57	-1.11	-0.75	-0.95	-0.68	-0.52	-	-1.35	-	-1.48
	Light	4.70	2.22	0.09	-0.04	-	-0.44	-1.11	-1.35	-0.91	-1.48	-0.84	-0.75	-	-1.05	-	-1.18
	Intense	3.96	2.00	0.17	-0.09	-	-0.35	-0.81	-1.65	-0.68	-1.00	-0.44	-1.11	-	-0.84	-1.18	-1.65
