# Supplementary material for: Disturbance regulates the density–body‐mass relationship of soil fauna
Source: Ecol Appl. 2019 Dec 2;30(1):e02019. doi: 10.1002/eap.2019 (PMC7003476; doi:10.1002/eap.2019)

Supporting Information. Frank van Langevelde, Vincent Comor, Steven de Bie, Herbert H. T. Prins, Madhav P. Thakur. 2019. Disturbance regulates the density–body mass relationship of soil fauna. *Ecological Applications*.

Appendix S3

Table S1: The density–body mass slopes obtained from Type II regression (RMA) matched the results from maximum likelihood estimates (MLE) for each sampling and treatment.

Treatment	Sampling	Slope (MLE)	Slope (RMA)
Control	3 weeks before	-0.99	-1.06
Light	3 weeks before	-0.99	-1.06
Intense	3 weeks before	-1.00	-1.05
Control	1 day after	-1.03	-1.06
Light	1 day after	-0.92	-0.95
Intense	1 day after	-0.72	-0.73
Control	1 month after	-1.04	-1.08
Light	1 month after	-0.79	-0.84
Intense	1 month after	-0.66	-0.69
Control	2 months after	-0.99	-1.06
Light	2 months after	-0.87	-0.95
Intense	2 months after	-0.74	-0.79

Figure S1: The body mass-density slopes (with standard errors) at different time points for disturbance treatments using Type I and Type II regressions.

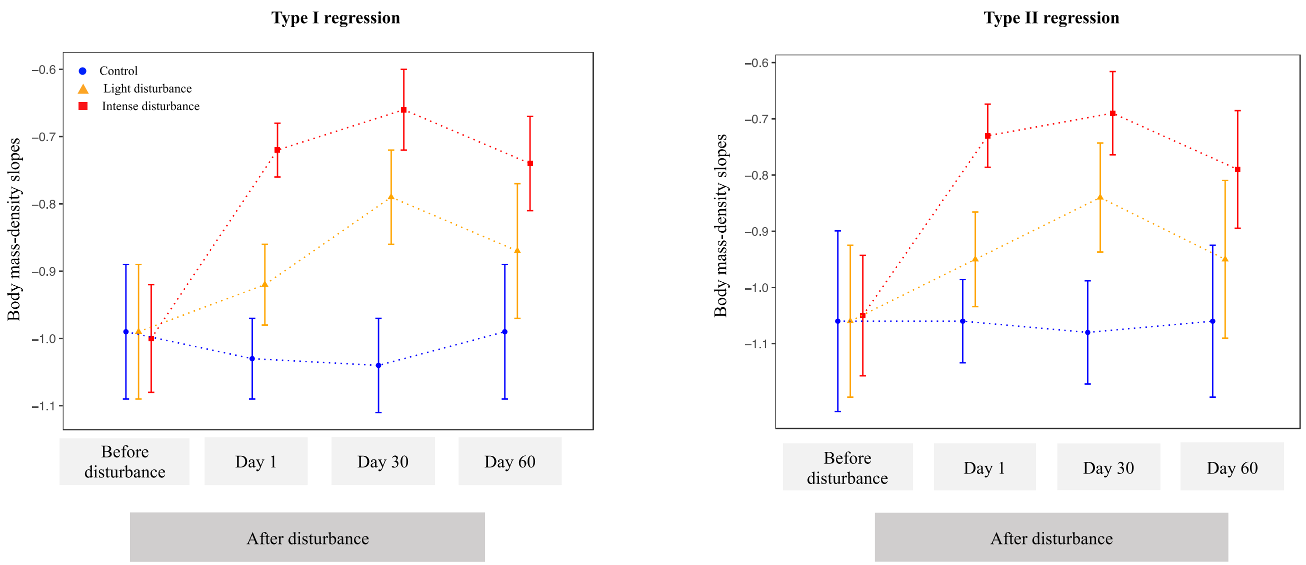

Supplement: Supplementary file 3 [file EAP-30-e02019-s003.pdf]
