# Supplementary material for: Disturbance regulates the density–body‐mass relationship of soil fauna
Source: Ecol Appl. 2019 Dec 2;30(1):e02019. doi: 10.1002/eap.2019 (PMC7003476; doi:10.1002/eap.2019)
Supplement: Supplementary file 4 [file EAP-30-e02019-s004.pdf]

Supporting Information. Frank van Langevelde, Vincent Comor, Steven de Bie, Herbert H. T. Prins, Madhav P. Thakur. 2019. Disturbance regulates the density–body mass relationship of soil fauna. *Ecological Applications*.

Appendix S4

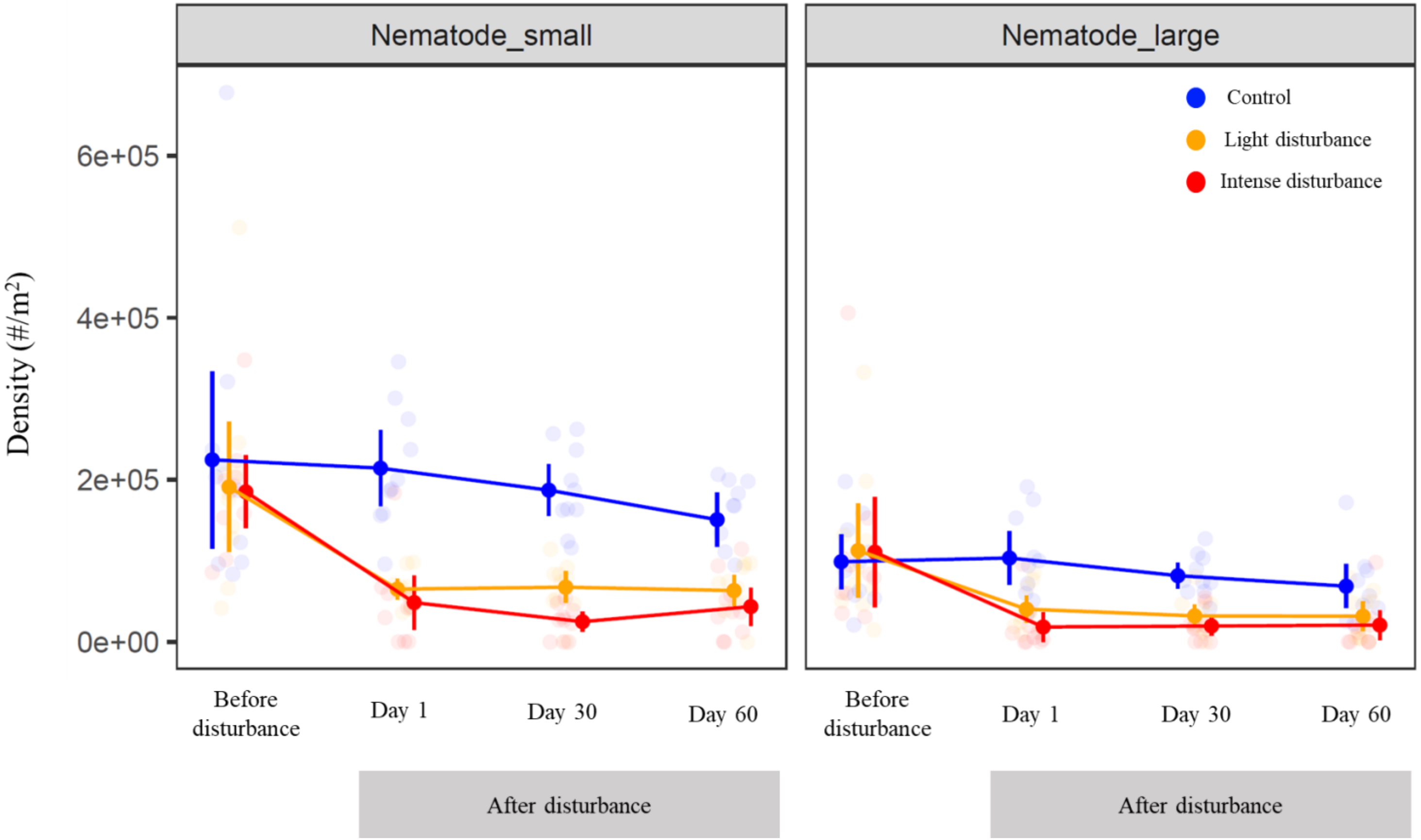

Figure S1: Density (mean ± standard error) of two size classes of nematodes during the experimental period in disturbance treatments.
